# Is suicide underreported? Evidence from Japan

**DOI:** 10.1007/s00127-021-02188-5

**Published:** 2021-11-12

**Authors:** Tetsuya Matsubayashi, Michiko Ueda

**Affiliations:** 1 Osaka School of International Public Policy, Osaka University, Osaka, Japan; 2 Waseda University, Tokyo, Japan

**Keywords:** Suicide, Mortality rate, Unknown causes, Underreporting, Japan

## Abstract

**Purpose:**

The underreporting of suicides has been a serious global concern among scholars and policymakers. Several studies have sought to detect the prevalence of underreporting by examining whether suicide mortality rates are negatively correlated with those due to unknown intent or causes. This study adds to the literature by examining the potential underreporting of suicides in Japan, where suicide rates have greatly declined in the recent years.

**Methods:**

We compiled subnational data from 47 prefectures between 1995 and 2016, obtained from Vital Statistics of Japan. We examined whether (1) mortality rates due to unknown intent or causes increased as suicide rates decreased; and (2) major socioeconomic causes of suicide (unemployment and divorce rates) had any relationship with the deaths due to unknown intent or causes.

**Results:**

Our analysis indicates that mortality rates due to unknown intent or causes were uncorrelated with suicide rates and the above socioeconomic indicators.

**Conclusions:**

In Japan, the frequency of suicides has no systematic relationship with deaths due to unknown intent or causes, suggesting the accuracy of suicide statistics.

## Introduction

Suicide is a serious public health concern worldwide. Scholars and policymakers have sought to identify the potential causes and effective prevention strategies of suicide, based on data obtained from suicide statistics. However, their accuracy has been questioned for many years (e.g., [1–5]). Suicides are likely to be underreported due to socio-cultural reasons, such as stigma and social pressures, and institutional reasons, such as the lack of resources for autopsy and rigorous classification of deaths [2, 6–10].

To understand the prevalence of underreporting in suicide statistics, scholars have developed several approaches and investigated whether and how likely suicides are misclassified as deaths due to other causes. The first approach (e.g., [9, 11]) uses detailed death records and assesses the process of classifying the cause of deaths by suicide. The second approach (e.g., [12, 13]) treats probable suicides (e.g., deaths by unknown causes or unidentified intent, and unintentional poisoning and drowning) as suicidal deaths and assesses whether alternative suicide rates including probable suicides are significantly higher than only those deaths which are classified as suicides in the original registry. The third approach (e.g., [14–16]) assesses whether suicides and probable suicides have similar background characteristics, such as gender, age, and medical history. The fourth approach (e.g., [6, 17–22]) assesses whether suicide rates are correlated with mortality rates due to other causes, such as unidentified injury, unidentified intent, unknown causes, or unintentional poisoning, under the assumption that suicidal cases are misclassified as deaths due to other causes, and thus, the underreporting of suicide results in the overreporting of deaths by these causes. Although the findings differ significantly across approaches and regions, overall, the underreporting of suicides is a frequently occurring, global problem [23].

This study adds to the literature by studying the potential underreporting of suicide in Japan. Our contributions are threefold. First, our focus was on Japan, where the potential underreporting of suicide has not yet been systematically examined. Japan is particularly an interesting case because the suicide rate has dramatically declined in the recent years from 40.0 per 100,000 population in 2002 to 23.2 in 2017 [24]; this decline may be partly attributable to underreporting. Second, we employed a regression-based approach developed by Kapusta et al. [6]. We used subnational data compiled from the Vital Statistics of Japan between 1995 and 2016, and examined whether suicide rates decreased as deaths due to unidentified injury, intent, or unknown causes increased in 47 prefectures during our study period. Our fixed-effect regression model allowed us to test whether suicide rates were negatively correlated with mortality rates due to unidentified intent or unknown causes after controlling for prefecture-specific time-invariant characteristics, time-specific shocks, and prefecture-specific time trends, thus eliminating the influences of major confounding factors, such as institutional environments and culture. Mortality rates were computed separately by age and sex. Third, we examined whether major socioeconomic causes of suicide, particularly unemployment and divorce rates, had any relationship with deaths due to unknown intent or causes. If a large number of suicide cases were coded as deaths by unknown causes or intent, the unemployment and divorce rates might be able to explain the change in deaths due to these causes. This analysis is motivated by the second approach mentioned above, which examines whether suicides and probable suicides share similar socioeconomic characteristics.

## Methods

The mortality data used in this study were obtained from death records preserved in the Vital Statistics of Japan. The data were collected for administrative purposes and anonymized and de-identified by the Ministry of Health, Labor and Welfare, before being released for research use. Individual death records were made available for research purposes by the Ministry. The vital statistics data are based on the death certificates issued by physicians and subsequently reported to the local government where the residency of the deceased is registered. The records cover all reported deaths in Japan, as well as those of Japanese citizens outside Japan. The records include the date of birth, date of death, reported place of death, and the underlying cause of death based on the International Classification of Diseases (ICD). Our study focused on the period between 1995 and 2016 because the ICD-10 standard became effective in 1995 in Japan; thus, we could use a consistent definition of deaths throughout our study.

Drawing on Kapusta et al. [6], we analyzed the data of deaths by suicide (X60–X84), unidentified intent (Y10–Y34), and unknown causes (R98 and R99). Note that we did not include deaths due to unknown causes coded as R00–R97 because the causes of deaths under these categories were partially known, though not fully determined. Moreover, their symptoms, such as symptoms and signs involving the circulatory and respiratory systems (R00–R09) and symptoms and signs involving the digestive system and abdomen (R10–R19), are rare in suicidal deaths. In Japan, all deaths due to external (or unnatural) causes, including the suspected cases, are reported to the police. Usually, a doctor assists the police in examining the circumstances to determine the cause of death before issuing a death certificate. Unless the cause of death is a suspected criminal activity, it is often determined without an internal examination. On an average, approximately 10% of deaths undergo internal examinations in Japan [25].

We excluded certain death records based on the following criteria: (1) Of individuals whose place of death was outside of Japan; (2) Records that did not include the date or place of death, because we will later aggregate the frequency of deaths by year and prefecture; and (3) Of individuals under the age of 19 because of the small number of suicide incidents in this age group.

We aggregated the individual death records by prefecture and year, producing panel data of mortality among 47 prefectures over 22 years. To consider the possibility that the underreporting of suicide was more prevalent among particular subpopulations, we tabulated the number of deaths for the above three causes by six age-sex groups: (1) men aged between 20 and 39 years, (2) men aged between 40 and 64 years, (3) men aged 65 years and over, (4) women aged between 20 and 39 years, (5) women aged between 40 and 64 years, and (6) women aged 65 years and over. We determined a mortality rate (the number of deaths per 100,000 population) for the six subgroups using population data from the Basic Resident Register for each year [26]. For each age-sex subgroup, the number of observations was 1034 (= 47 prefectures × 22 years). The unit of observation was prefecture-year.

Using the mortality rate data of suicide, unidentified intent, and unknown causes in 47 prefectures between 1995 and 2016 for each of the six age-sex groups, we first examined whether suicide mortality rates were negatively correlated with those due to unidentified intent or unknown causes. We formulated the following model:

1 $$\left[ {{\text{Suicide}}} \right]_{jt} = \beta_{1} \left[ {\text{Unidentified intent}} \right]_{jt} + \beta_{2} \left[ {\text{Unknown cause}} \right]_{jt} + \gamma_{j} T + \varphi_{t} + \rho_{j} + \varepsilon_{jt}$$

where ${[\mathrm{Suicide}]}_{jt}$, ${[\text{Unidentified intent}]}_{jt}$, and ${[\text{Unknown cause}]}_{jt}$ each denote a mortality rate per 100,000 population. If suspicious suicidal cases were coded as deaths by unidentified intent or unknown causes, ${\beta }_{1}$ and ${\beta }_{2}$ would be estimated to be negative, which would then suggest the potential underreporting of suicide.

In Eq. (1), φ_*t*_ represents the year fixed effects and ρ_*﻿j*_ represents the prefecture fixed effects unique to each prefecture, while γ_*﻿j*_*T* represents the prefecture-specific linear time trends. The inclusion of the year fixed effects allowed us to control for the influences of annual socioeconomic fluctuations at the national level, such as the macroeconomic policies and business cycles, which might affect the mortality rate in the entire country. On the other hand, the inclusion of the prefecture fixed effects allowed us to control for the effects of time-invariant characteristics of prefectures, such as social norms related to suicide, climate and geographical conditions, and relatively stable autopsy-related practices. Furthermore, the inclusion of γ_*﻿j*_*T* in the model allowed us to consider the linear (typically declining) trends in suicide rates unique to each prefecture. All these terms are crucial in our estimation because we aimed to isolate the relationship between suicide rates and mortality rates due to unidentified intent or unknown causes after controlling for these major potential confounders. Moreover, to account for the potential serial correlation in the error term within the prefectures and heteroskedasticity, the standard errors were clustered by prefecture.

Next, we examined whether the major socioeconomic causes of suicide (i.e., unemployment and divorce rates) had any relationship with mortality rates due to unknown intent or causes. We modified Eq. (1) as follows:

2 $$\left[ {{\text{Mortality}}} \right]_{jt} = \beta_{1} \left[ {{\text{Unemployment}}} \right]_{jt} + \beta_{2} \left[ {{\text{Divorce}}} \right]_{jt} + \gamma_{j} T + \varphi_{t} + \rho_{j} + \varepsilon_{jt} ,$$

where the outcome variable ${[\mathrm{Mortality}]}_{jt}$ denotes either ${[\mathrm{Suicide}]}_{jt}$, ${[\text{Unidentified intent}]}_{jt}$, or ${[\text{Unknown cause}]}_{jt}$. ${[\mathrm{Unemployment}]}_{jt}$ denotes the unemployment rate in percentages in prefecture *j,* in year *t*, and ${[\mathrm{Divorce}]}_{jt}$ denotes the divorce rate per 1000 persons. They were obtained from the System of Social and Demographic Statistics of Japan [27]. The data of the unemployment rate in the prefectures were available only from 1997; therefore, we limited our analysis between 1997 and 2016. Thus, here, the number of observations was 940 (= 47 prefectures × 20 years). The standard errors were clustered by prefecture.

These two socioeconomic variables are the major predictors of suicide worldwide (e.g., [28]). We anticipated that the deaths due to unidentified intent and unknown causes share the same underlying mechanism as suicides. Therefore, we analyzed whether the unemployment and divorce rates were positively correlated with the suicide rates in our data, and a similar relationship existed with the mortality rates due to unidentified intent and unknown causes. If the unemployment and/or divorce rates have a positive relationship with the mortality rates due to unidentified intent and unknown causes, it suggests that the classification of deaths due to unidentified intent and unknown causes might include some suicidal cases; thereby indicating the underreporting of suicides. Equations (1) and (2) were estimated using the ordinary least squares estimator and Stata software version 15 (Stata Corporation).

## Results

Table 1 presents the total number of deaths due to suicide, unidentified intent, and unknown causes, for the six age-sex groups between 1995 and 2016 in Japan. In addition, it reports the average population size of each subgroup between 1996 and 2016 to calculate a mortality rate during the study period. Table 2 reports the summary statistics of these variables. Suicide was reported as a cause of death more frequently than unidentified intent and unknown causes among all six age-sex groups. The relative frequencies of deaths due to unidentified intent and unknown causes was greater among middle-aged and older men and women. The relative size of deaths due to unknown causes among men and women aged 65 and over was particularly large.

**Table 1:** The total number of deaths due to suicide, unidentified intent, and unknown causes in Japan (1995–2016)

Age groups	Men			Women		
	20–39	40–64	65 and over	20–39	40–64	65 and over
Suicide	101,461	222,104	103,360	40,439	68,725	68,637
Unidentified intent	5,661	13,491	9,264	2,884	5,260	7,084
Unknown causes	5,386	35,820	45,251	2,119	7,565	30,898
Average population size between 1995–2016	17,006,334	21,588,614	10,796,533	16,303,056	21,644,681	14,737,517

Source: The Vital Statistics and the Basic Resident Register

**Table 2:** Summary statistics

	Mean	S.D.	Min	Max
Mortality rate by suicide, men 20–39	28.922	7.095	10.053	57.744
Mortality rate by suicide, women 20–39	10.579	3.034	1.231	21.551
Mortality rate by suicide, men 40–64	49.587	14.223	21.245	108.217
Mortality rate by suicide, women 40–64	14.184	2.978	5.534	25.202
Mortality rate by suicide, men 65 and over	45.952	11.531	16.862	103.074
Mortality rate by suicide, women 65 and over	22.397	8.187	4.172	66.824
Mortality rate by unidentified intent, men 20–39	1.488	1.229	0.000	9.479
Mortality rate by unidentified intent, women 20–39	0.721	0.758	0.000	5.663
Mortality rate by unidentified intent, men 40–64	2.794	2.022	0.000	16.068
Mortality rate by unidentified intent, women 40–64	1.090	0.958	0.000	6.178
Mortality rate by unidentified intent, women 65 and over	3.543	3.498	0.000	26.524
Mortality rate by unidentified intent, women 65 and over	2.033	1.836	0.000	14.586
Mortality rate by unknown causes, men 20–39	1.299	1.181	0.000	13.026
Mortality rate by unknown causes, women 20–39	0.505	0.664	0.000	5.987
Mortality rate by unknown causes, men 40–64	6.710	5.929	0.000	36.540
Mortality rate by unknown causes, women 40–64	1.424	1.447	0.000	7.954
Mortality rate by unknown causes, men 65 and over	14.908	17.712	0.000	139.031
Mortality rate by unknown causes, women 65 and over	7.895	10.139	0.000	85.873
Population size, men 20–39	361,837	396,000	61,283	2,068,652
Population size, women 20–39	346,874	366,907	58,771	1,909,322
Population size, men 40–64	459,332	438,982	93,039	2,346,442
Population size, women 40–64	460,525	428,776	94,233	2,248,188
Population size, men 65 and over	229,713	204,055	45,684	1,298,820
Population size, women 65 and over	313,564	263,006	71,277	1,700,257
Unemployment rate (%)	4.027	1.089	1.700	8.400
Divorce rate per 1000	1.849	0.305	1.060	2.940

Table entries are the mortality rates for each cause of death per 100,000 population, population size, the unemployment, and divorce rates. The number of observations is 1034 (= 47 prefectures × 22 years) for the mortality-related variables and 940 (= 47 prefectures × 20 years) for the unemployment and divorce rates. The unit of observation is prefecture-year

**Fig. 1:**
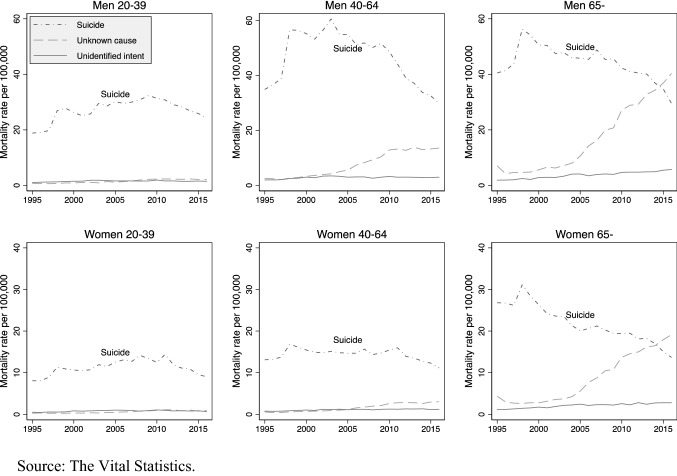
Mortality rates due to suicide, unidentified intent, and unknown causes by sex and age group in Japan, 1995–2016.

Figure 1 displays the temporal changes in mortality rates by three types of deaths among the six age-sex groups and shows several interesting patterns. First, as mentioned above, the decline in suicide rate in the last decade was consistent across all the age-sex groups, but most evident among men aged 40–64 years, and both men and women aged 65 years and above. Second, these groups experienced a modest to large increase in mortality rates due to unknown causes after 2005. Notably, the mortality rates due to unknown causes exceeded those by suicide in 2015 among the elderly population, regardless of sex. Third, the mortality rates due to unidentified intent exhibited little change for all the age-sex groups between 1995 and 2016. Overall, the decrease in the suicide rates and the increase in the mortality rate due to unknown causes among some groups suggests that some suicide deaths could have been coded as death due to unknown causes.

However, the above observations do not consider any time- or area-specific characteristics and changes. To test this possibility in a more rigorous manner, Table 3 reports the estimation results of Eq. (1), where the suicide rates were regressed on the mortality rates due to unidentified intent and unknown causes. The prefecture and year fixed effects and the prefecture-specific linear time trend were included in the estimation, but their coefficients and standard errors were not reported. According to Table 3, there was no statistically significant relationship between the mortality rates due to unidentified intent and unknown causes and the suicide rates across all the age-sex groups.

**Table 3:** The relationship of suicide rates with mortality rates due to unknown causes or unidentified intent in 47 prefectures in Japan (1995–2016)

	Men			Women		
	20–39	40–64	65 and over	20–39	40–64	65 and over
Unidentified intent	0.134	0.031	0.031	0.249	− 0.039	0.006
	(0.180)	(0.048)	(0.048)	(0.225)	(0.119)	(0.034)
Unknown causes	− 0.016	0.088	0.088	0.048	− 0.087	− 0.078
	(0.228)	(0.099)	(0.099)	(− 0.191)	(0.162)	(0.113)
*R*^2^	0.508	0.785	0.568	0.362	0.346	0.693
*N*	1034	1034	1034	1034	1034	1034

Table entries are linear regression estimates with standard errors in parentheses. The unit of observation is prefecture-year. The standard errors are clustered by prefecture. The outcome variable was the suicide rate per 100,000 people. All models include prefecture, year fixed effects and prefecture-specific linear time trends. *p < 0.10, **p < 0.05, ***p < 0.01 (two-tailed tests)

Table 4 reports the estimation results of Eq. (2), where the mortality rates due to the three causes of death were regressed on the unemployment and divorce rates. Similar to Table 3, the results of the prefecture and year fixed effects and the prefecture-specific linear time were not reported. Panel (1) of Table 4 focuses on the suicide rates as an outcome variable. As expected, the unemployment and divorce rates had a positive and statistically significant relationship with the male suicide rates. As the unemployment rate increased by 1 percent, the suicide rate per 100,000 increased by 1 among men aged 20–39, 4.2 among men aged 40–64, and 2.6 among men aged 65 and over. As the divorce rate increased by 1 per 1000 population, the suicide rate increased by 10 among young and senior men. Contrastingly, we found no significant relationship between these two socioeconomic indicators and female suicide rates.

**Table 4:** The relationship between two measures of socioeconomic characteristics and mortality rates due to suicide, unidentified intent, and unknown causes in 47 prefectures in Japan (1997–2016)

(1) Suicide	Men			Women		
	20–39	40–64	65 and over	20–39	40–64	65 and over
Unemployment rate	1.018*	4.191***	2.570***	− 0.129	0.194	0.564
	(0.564)	(0.957)	(0.903)	(0.277)	(0.272)	(0.532)
Divorce rate	10.343***	14.615	10.644*	0.494	1.437	− 3.892
	(3.435)	(9.212)	(5.827)	(1.942)	(1.800)	(3.119)
*R*^2^	0.410	0.811	0.595	0.362	0.346	0.693
*N*	940	940	940	940	940	940

(2) Unidentified intent	Men			Women		
	20–39	40–64	65 and over	20–39	40–64	65 and over
Unemployment rate	− 0.120	− 0.127	− 0.086	− 0.010	− 0.019	− 0.297
	(0.099)	(0.192)	(0.321)	(0.084)	(0.104)	(0.207)
Divorce rate	0.933	0.442	− 1.053	− 0.571	− 1.224***	− 1.735
	(0.776)	(1.035)	(1.341)	(0.509)	(0.401)	(1.037)
*R*^2^	0.202	0.363	0.537	0.140	0.279	0.370
*N*	940	940	940	940	940	940

(3) Unknown causes	Men			Women		
	20–39	40–64	65 and over	20–39	40–64	65 and over
Unemployment rate	− 0.115	0.032	1.670	0.049	0.003	0.472
	(0.095)	(0.404)	(1.253)	(0.061)	(0.131)	(0.618)
Divorce rate	0.492	4.282**	8.659	− 0.554	1.248	4.805
	(0.920)	(1.954)	(6.861)	(0.344)	(0.765)	(3.517)
*R*^2^	0.319	0.826	0.875	0.275	0.600	0.857
*N*	940	940	940	940	940	940

Table entries are linear regression estimates with standard errors in parentheses. The unit of observation is prefecture-year. The standard errors are clustered by prefecture. The outcome variables are the mortality rates per 100,000 population by suicide in panel (1), unidentified intent in panel (2), and unknown causes in panel (3). All models include prefecture and year fixed effects and prefecture-specific linear time trends. *p < 0.10, **p < 0.05, ***p < 0.01 (two-tailed tests).

Based on these results, we further tested whether these socioeconomic indicators were also strong predictors of the mortality rates due to unidentified intent and unknown causes. If some of the deaths coded as due to unidentified intent and unknown causes were actually suicide cases, we should be able to find a strong positive relationship between the major predictors of suicide and the mortality rates due to unidentified intent and unknown causes, especially among men. Panels (2) and (3) of Table 4 show the estimation results, where the mortality rates due to unidentified intent and unknown causes are the outcome variables. We found no statistically significant relationship between these causes and the sociodemographic indicators, except between the divorce rate and mortality rate due to unknown causes among men aged 40–64.

## Discussion

This study examined the possibility that suicide is underreported in Japan. Using prefecture-level data between 1995 and 2016, we examined whether suicide rates were negatively correlated with mortality rates due to unidentified intent or unknown causes. Our analysis found that suicide rates decreased rapidly among middle-aged and elderly populations and the mortality rates due to unknown causes increased rapidly among senior men and women in recent years of our study period. However, the results of our fixed-effect regression indicated that there was no significant relationship between these mortality rates. Thus, once we consider the effects of various underlying factors, there is no statistically significant relationship between the mortality rates due to suicide and unknown causes, suggesting that it is unlikely that suicidal cases were recoded and counted as deaths due to unidentified intent or unknown causes. Moreover, unemployment and divorce rates had a significant, positive relationship with male suicide rates; no such relationship was found with the mortality rates due to unidentified intent and unknown causes. Thus, these results offer little evidence for the possibility that suicidal cases were classified as deaths due to unidentified intent or unknown causes.

We checked the robustness of our findings in several ways. First, we included the mortality rate by accidents, including traffic accidents and drowning, as another right-hand side variables and estimated Eq. (1). The results were generally consistent with those in Table 3; the mortality rate due to accidents had no significant relationship with suicide rates. Second, we confirmed that the results reported in Table 3 held true even after unemployment and divorce rates were included on the right-hand side of Eq. (1). Third, we also confirmed that the mortality rates transformed into a natural log did not change the results.

Our findings offer no strong evidence for the possibility of underreporting suicidal deaths in the Japanese context. Varnik et al. [21] proposed that suicide statistics can be considered valid if the mortality rate due to unidentified intent is below 2.0/100,000 and the proportion of deaths due to unidentified intent to suicide is below 20%. According to this 2–20 benchmark, the suicide statistics in Japan is mostly valid (see Table 2).

This study has a couple of limitations. First, most importantly, our findings cannot be viewed as direct evidence to show that suicides are not underreported in Japan. We only showed that suicidal deaths were unlikely to be recorded as deaths due to unidentified intent and unknown causes, and that the two major determinants of suicide were uncorrelated with deaths due to unidentified intent and unknown causes. To reject the possibility of underreporting of suicide, we need to rely on a more direct approach, such as examining detailed individual death records categorized as deaths by accidents, unidentified intent, or unknown causes, and assess the process of classification. Second, our data excluded death records without information on the date or place of death. This might result in the undercount of deaths from unknown causes or intent because the cause of death was more likely to be unidentified when the details of deaths were unknown.

## Declarations

### Availability of data and material

The approval to use the Vital Statistics for academic research was obtained from the Ministry of Health, Labor, and Welfare (MHLW) by Haruko Noguchi and Akira Kawamura under Tohatsu-0507-3.The data are not available for public use because the death records from the Vital Statistics of Japan in this study were made available by the Ministry of Health, Labor and Welfare of Japan for research use upon request.

### Code availability

Available upon request.
